# Moral craft: engaging with value pluralism in healthcare decision-making

**DOI:** 10.1007/s40592-025-00266-x

**Published:** 2025-10-13

**Authors:** Michael Parker

**Affiliations:** Ethox Centre, University of Oxford, Oxford, United Kingdom

**Keywords:** Healthcare ethics, Medical decision-making, Moral craftsmanship, Professional ethics, Value conflict, Value pluralism

## Abstract

Healthcare professionals routinely navigate complex value conflicts that span personal, interpersonal, and organisational domains. This paper examines the concept of moral craftsmanship—the skilled practice of understanding, analysing, and working through value conflicts in healthcare settings—and argues that value pluralism provides a more realistic framework for healthcare ethics than approaches seeking overarching moral consensus. Through analysis of cases spanning clinical genetics, paediatric end-of-life care, and institutional resource allocation, the paper explores how value conflicts manifest across interconnected domains and explores the practical reasoning processes through which healthcare professionals successfully navigate seemingly intractable moral disagreements. Drawing on examples from clinical genetics counselling and recent analyses of dissensus in paediatric care, the paper argues that deep value pluralism is compatible with reasoned decision-making and that moral craftsmanship represents an essential skill for effective healthcare practice. Oversimplified ethical frameworks risk creating dangerous gaps between institutional processes and lived moral experience, potentially undermining public trust in healthcare systems. Healthcare institutions must develop approaches that acknowledge genuine value plurality while supporting practical decision-making, maintaining mechanisms for incorporating diverse public values, and addressing the moral residue that persists beyond immediate decisions.

## Moral craftsmanship

Moral craftsmanship is central to the work of the health professional. In their care for patients and families, clinicians are required to undertake a significant amount of what I have referred to elsewhere as ‘moral work’. (Parker 2012) Much of this work is not visible, and in many cases would not be thought of as such by health professionals themselves or by their patients. Take for example, the following hypothetical case in the context of clinical genetics.

Rachel is a woman in her early twenties who has recently come to know about a significant history of breast cancer in her family. She is very anxious about the possibility of having inherited this risk from her mother who died from the disease at a young age. When she comes to the genetics clinic for her appointment with a genetic counsellor to discuss a test Rachel mentions that she is an identical twin and that her sister, Michelle, is very much against the test. She will not take a test herself and does not want Rachel to do so either. Michelle is adamant that she does not want to know her disease risk status. Because they are identical twins, a test on Rachel would also be a test on Michelle.

In this case as in many other areas of healthcare, good practice requires the health professional to care for patients and families in the context of conflicting values. Even though it would be an unusual health professional who referred to this explicitly as ‘moral work’, most are well used to this kind of task and experienced professionals and teams will have developed effective approaches to such problems. Work of this kind is an enduring feature of the day-to-day moral craftsmanship of health professionals, and a core component of good practice. Through it they are often successful at finding ways for their patients, in this case the sisters, to navigate towards solutions that take their competing values seriously. In this case, finding a way forward might be expected to involve conversations with each sister individually to explore concerns and possible ways forward, offering support with communication between them, allowing time for reflection, and so on. Often this work will be successful. In some situations, however, despite their best efforts, competing moral values are not amenable to this kind of work and the health professional’s moral work fails to provide an effective way of navigating the value conflict. In some cases, this will be because of an unwillingness or inability of the relevant parties to engage in productive dialogue. In others, however, it will be because the value conflict is deeper or more resistant to such work. These more intractable kinds of situations are ones in which it would be common for health professionals to describe themselves as confronted by an ‘ethical dilemma’. It might be the kind of ‘case’ that they would refer to an ethics committee or be motivated to call upon the expertise of an ethicist. These are cases that call for a different kind of work, a form of moral craftsmanship more commonly and explicitly considered by health professionals to be ‘ethics’. In the case above, for example, if no way forward can be found, the health professional is confronted with a seemingly intractable conflict between on the one hand conducting a genetic test on a patient without her consent and on the other refusing to conduct a test on a patient who wishes to have it as a way of managing her risk.^1^ And this is clearly an ethical problem.

This brief example illustrates that the professional lives of health professionals are complex and multifaceted moral worlds of which moral craftsmanship constitutes an important aspect. Rapid technological innovation, constrained resources, and an increased focus on patient-centredness, mean that such conflicts and the requirement for moral craftsmanship—whether moral work or ethics - are more common and of greater intensity.

## Domains of value conflict in healthcare (and the interactions between them)

Value conflicts in healthcare requiring one or other of these complementary forms of moral craftsmanship might usefully be thought of as arising in three interconnected and overlapping domains: the personal, the interpersonal, and the organisational. Many of the most interesting value conflicts are those that cut across these domains.

### Personal

The least visible but perhaps most intensely felt value conflicts are those experienced ‘internally’ by individuals who are personally committed to competing values and need to make decisions in which no available course of action can satisfy them all. In such situations, value conflict can take the form of an on-going internal struggle. We have all have experienced such struggles. They are an unavoidable feature of what it means to live a human life (Berlin 1969). In the context of healthcare decision making personal value conflicts of this kind can be particularly difficult. Many healthcare decisions are experienced by patients, or potential patients, as having an important moral dimension. The decision about whether or not to terminate a pregnancy, about whether to donate an organ, to accept a blood transplant, or how best to break bad news, are all paradigmatic examples in the bioethics literature, and in the training of clinicians. Supporting patients as they seek to understand, work through and resolve such value conflicts is an important part of the work of many health professionals whose role involves interactions with patients. These are situations that arise out of being human beings with competing values, commitments, and priorities. This is, of course, as true of health professionals as it is of patients. Many health professionals will at some point in their career struggle with the making of decisions with regard to which they have conflicting commitments or values, and in some cases this will raise the question of conscientious objection.

### Interpersonal

Interpersonal value conflict, by contrast, involves moral disagreement between people. These too are a central concern of much debate in medical ethics and can take many forms. Three of the most important of these are: disagreements between family members (such as that between Rachel and Michelle above); disagreements between patients (or families) and health professionals; and disagreements between health professionals or within clinical teams. Each of these kinds of interpersonal moral disagreement can arise across a wide range of healthcare settings and domains. Value disagreements between patients are not uncommon and can be particularly intense in areas of medical practice in which bioethics has an interest: end of life decision making, reproductive medicine, genetic testing and so on. An area of medicine where interpersonal value conflicts of this kind can be particularly difficult is in end of life decision making for children, where parents disagree. Here is a fictional example.

Marina and Stefan are the parents of an infant born very prematurely. Their baby, Layla, has a brain haemorrhage and is dependent on mechanical ventilation. The doctors believe that Layla will have profound cognitive and physical impairments if she survives. Stefan believes that all life is sacred and that any chance of survival is worth pursuing. Marina views quality of life as more important and believes that withdrawing ventilator support and allowing Layla to die would be the more humane path given her injuries. The parents have radically different values with respect to Layla’s care and about whether to transition to palliative care. The healthcare team members are struggling to navigate this value disagreement whilst ensuring that Layla’s interests remain central.

This is an example of a situation in which parents disagree with each other. Value conflict between patients and health professionals is also not uncommon. In situations similar to the one above, for example, parents might have a shared view that is in conflict with that of the health professionals. The most difficult cases tend to be those in which health professionals consider it to be in the interest of a child for life sustaining treatment to be withdrawn but parents want everything done, where ‘everything’ includes major interventions with very low chance of success. When such cases come to law they tend to be very high profile. In cases like these, value conflict can also occur between health professionals. Clinicians may themselves be divided between those who believe a child such as Layla’s best interests lie in withholding treatment and those who take the view that there is a case to be made for further intervention (Wilkinson and Savulescu 2019). In reality, of course, it is likely that many of those involved in such cases will, as individuals, be struggling personally ‘internally’ with competing values and experiencing a significant degree of personal value conflict, and moral distress.

### Organisational

Some value conflicts, whilst being informed by and shaping the arguments and decisions of individual health professionals and patients and the relationships between them, might perhaps be best considered organisational. Value conflict in the organisational domain is also a staple of contemporary writing in bioethics. In some cases, such conflicts will arise because of competing organisational policy options, priorities or remits. Health institutions, whether health systems or individual hospitals, must deal with competing priorities with a value component in more or less every aspect of their activity. The most obvious example is the need to make decisions about the allocation of scarce resources. Other examples will include the development of policies about the sharing of data, the deployment of public health powers and so on.

### Connectedness between the levels

Although distinctions between these different levels are useful when analysing complex value disagreements, it is clear that many of the most interesting and difficult value conflicts in healthcare will reach across these domains. Value conflicts will often, perhaps even mostly, be characterised by multiple interconnections and interdependencies between individuals, groups, and organisations. An important example in the context of publicly funded health care (but also in relation to health insurance coverage) is where individuals believe that health resources, for example expensive drugs or other treatments, should be (or should have been) allocated differently. Here there are likely to be differences of value at the personal, interpersonal, and organisational levels, and between them. Others might be situations in which there are explicitly moral conflicts between what a health system does and what individuals or groups believe it ought to do: e.g. disagreements about the provision of contraceptive advice or puberty blockers to teenagers. Even conflicts which do not initially look organisational can develop to become problems across all of these areas. A good example of this in the UK is the Charlie Gard case which is discussed in more detail below. Healthcare practice is constituted by clusters of overlapping and interconnected domains in which deep value conflict is pervasive and resistant to resolution through appeal to an agreed overarching value.

## Value pluralism and decision making

What are the implications of taking seriously both the existence of pervasive value pluralism in healthcare and the practices of moral craftsmanship through which health professionals and patients navigate value conflict successfully? While taking value pluralism seriously accords well with moral experience, it presents a difficult problem with respect to decision making and raises questions about what might be achieved through moral craftsmanship. For, if many value disagreements in healthcare—the ones we are most interested in as bioethicists - cannot be reconciled by appeal to an overarching value, that is, if values are fundamentally plural and incommensurable, what does this mean for the possibility of reasoned moral choices? Is its implication that, even though they might not be experienced as such, choices in the face of incommensurable values are in effect merely instances of *plumping* for one option rather than another? How, if at all, against this background of deep value plurality and radical moral disagreement might reasoned decision making about competing values be conceptualised as possible?

It is important to note, as an aside, that in many areas of healthcare, formal structures are in place to enable the making of decisions in the face of conflicting values. This is sometimes because there is guidance, or legal clarity about whose values should be prioritised. For example, the legal right in the UK for competent adult patients to refuse even potentially life-saving treatment means that in such cases health professionals know what they must do. In other situations, as described above, progress in the face of different values may be possible because the successful moral work of health professionals enables people who begin with seemingly conflicting values—through a process of deliberation, discussion and reflection involving supportive efforts—to find values in common or reach agreement on a way forward that respects what looked previously to be irreconcilable positions.

Whilst the law can provide answers to some value conflicts and the moral work of health professionals can navigate consensual pathways through others, this is not always possible, and is unlikely to be achievable in many of the most difficult cases. Value conflict can persist even if the people involved have support and opportunities to discuss the decision with friends and health professionals, and even where all are well-intentioned and motivated to reach a solution. Such value conflict is a pervasive feature of human experience, and healthcare practice is a space in which they can be particularly intense. It is also, however, a domain in which decisions need to be made- by patients, health professionals, clinical teams, and organisations—often against a backdrop of deep moral disagreement and often with great urgency. To what extent is it coherent to have both a strong sense that the moral world is characterised by deep value conflicts and to have reason to believe there to be the possibility of reasoned decision-making in the face of incommensurable values?

### The compatibility of value pluralism with reasoned decision making

Making difficult decisions between competing and often incommensurable values is a feature of many people’s lives for much of the time. There seems no good reason to believe that value pluralism is incompatible with reasoned decision making and much to suggest that it is. Michael Stocker provides and analyses an impressive range of such cases in his book ‘Plural and conflicting values’ (Stocker 1990). Here is an anonymised composite example of my own based on experience from my work with genetics professionals on the ethical aspects of their work (Parker 2012).

A woman with a deeply held religious commitment has a young child with a developmental disorder requiring a very high level of support from herself, her family, and from medical professionals. She is very devoted to her child’s care and has given up working to focus on this. During the early stages of a second pregnancy, the woman is informed that this pregnancy is also affected by the same developmental disorder. The woman is very strongly of the view that termination of pregnancy is immoral. However, after a profoundly difficult and emotionally draining period of reflection she decides to terminate the pregnancy. She believes that she would not be able to provide good quality support to two children if she were to proceed with the pregnancy. She feels deeply upset about her decision and has some regrets, but she feels she has made the right choice.

This case and others like it illustrate the possibility of thoughtful, carefully considered moral choice in the face of deeply difficult moral complexity and value conflict at the personal level. This case also illustrates another important advantage value pluralism has in that it reminds us that the making of a morally difficult decision is not usually the end of the moral matter. Regret, sympathy, and practical support can make moral sense and be required. This question of moral residue of these and other kinds is insufficiently explored in bioethics in practice, which tends to be overly focused on narrow concepts of moral reason and justification.

It is also possible for bioethics at the interpersonal and institutional levels to both take value pluralism seriously and have a reasoned approach to decision making. An interesting recent example of an attempt to achieve this is Dominic Wilkinson and Julian Savulescu’s analysis of the Charlie Gard case in their book, Ethics, Conflict and Medical Treatment for Children, (Wilkinson and Savulescu 2019). In the book, they provide several examples of difficult and troubling value conflicts arising in relation to the care of seriously ill children. The central case they discuss is that of Charlie Gard, an infant with a rare genetic condition that meant he was very unlikely to survive past his first birthday^2^. Charlie’s parents wished to take him from the hospital in the UK and fly him to the United States for an experimental treatment. The hospital team caring for him believed this to be against his best interests because in their view it would extend his suffering with no realistic chance of success. With this in mind, the medical team went to court to prevent this course of action. There were, however, differences of opinion among the clinical team. Inspired by this case, and by other similar cases in which there is disagreement within the clinical team about end of life decision making i.e. situations in which some clinicians are willing to treat - or in this case to support and facilitate the transfer of the child to another hospital for treatment—Wilkinson and Savulescu provide an argument that, contrary to current guidelines, the requirement for the achievement of consensus among health professionals in cases involving decisions about treatment limitation should be dropped. They argue that ‘instead of trying to achieve agreement… professionals should acknowledge and accept disagreement in ethically complex decisions’ (Wilkinson and Savulescu 2019, p. 103) They hold that as long as the decision would be lawful, dissensus, which here requires the existence of a range of different professionally appropriate considered judgements, should be enough to justify decisions within this range. And that, in practice,

‘Treatment provision [or limitation] may be discussed with families and may proceed where at least one member of the treating team who is aware of the relevant clinical facts, after adequate reflection and discussion, would endorse such a decision (and be willing to take over the care of the patient if they are not the responsible clinician) (Wilkinson and Savulescu 2019, p. 106).

In their book length deliberation on the requirements for good decision making in such cases, Wilkinson and Savulescu provide a powerful illustration of the possibility of reasoned and non-reductive resolution of interpersonal and institutional value conflict in practice without appeal to a master value or an elision of conflicting moral concerns. Other processes could have been chosen. However, what Wilkinson and Savulescu have provided, I think, is evidence that deep value pluralism and value conflict are not incompatible with reasoned decision making at the interpersonal and organisational level.

Taken together, these two examples—the case above and Wilkinson and Savulescu’s discussion of dissensus—provide reason to believe that through the application of practical reason it is possible to make situated and contextually relevant, considered judgements and decisions in the face of radical value conflict without appeal to ethical frameworks or an overarching master value.

## Conclusion

I began this paper by mapping out some of the different ways in which value conflict is a feature of healthcare practice, emphasising the overlapping and interconnected features of these problems. Value conflicts in healthcare practice and policymaking take place in complex and heterogenous moral landscapes. An adequate bioethical analysis will be one that is capable of acknowledging the irreducibility of many value conflicts, paying careful attention to the interconnections between the personal, interpersonal, and institutional domains, and focusing on thoughtful practical judgement.

When healthcare systems—and medical research institutions—adopt oversimplified frameworks for handling value conflicts, they risk creating a dangerous gap between institutional processes and the lived moral experience of patients, families, and healthcare workers—they fail to do justice to the moral experience of those involved. Patients and families whose complex moral struggles are reduced to what can be experienced as formulaic ethical principles may feel that their genuine moral concerns have been dismissed or misunderstood. Healthcare professionals forced to operate within oversimplified ethical frameworks may experience moral distress when they cannot acknowledge or address the full complexity of situations they face. This gap threatens both public trust, system sustainability, and health system resilience. Careful attention needs to be paid to work aimed at ensuring that they don’t drift too far apart because of the threat this poses to the possibility of well-founded public trust and confidence required for trustworthy healthcare systems grounded in commitments to social justice. The sustainability of healthcare systems, particularly those grounded in social justice like the United Kingdom’s NHS, depends on maintaining what might be thought of as a ‘social contract’ between healthcare institutions and the publics they serve (Lucassen et al. 2017). This contract requires that institutional approaches to handling value conflicts remain connected to and respectful of the way moral values are experienced and negotiated in personal and professional life. When ethical frameworks and the institutional processes they inform become too detached from this lived moral experience, they risk eroding the conditions for well-founded public trust. One implication of the above is that careful attention should be paid to developing and implementing mechanisms for incorporating public values into healthcare and to ensuring—as far as possible—that the value basis for the conditions capable of supporting well-founded public trust and confidence is in place. In this context, reflective equilibrium about the conditions for a coherent and sustainable relationship between public and private values is of moral importance in itself, and for social justice. (Williams 1981, p. 82)

Taking value pluralism seriously requires approaches that can better acknowledge and work with value conflict while still supporting practical decision-making. This means: developing models that can acknowledge genuine value plurality without falling into paralysis; creating institutional processes that can recognize and support engagement with moral complexity; maintaining mechanisms for incorporating diverse public values into healthcare decision-making; and acknowledging and addressing moral residue that persists beyond immediate decisions. It also implies a central role for moral craftsmanship in medical practice and the need for its cultivation in medical student training. Such approaches, while more complex and possibly more labour intensive in the short term than other available ways of doing bioethics, are essential for maintaining the conditions of trust and legitimacy required for sustainable healthcare systems. They also help to foreground the central importance of moral craftsmanship in effective and ethical health systems.

Like human life more broadly, healthcare and the related worlds of medical research and technology development are characterised by sometimes incommensurable value differences. Much contemporary bioethics fails to take this deep value pluralism sufficiently seriously and fails to capture the richness and complexity of moral experience. There is a need for richer value pluralistic accounts of healthcare ethics that acknowledge genuine incommensurability of values and gives a central role to the skills of empathic, often relational practical moral reason and moral craftsmanship.

## Data Availability

No datasets were generated or analysed during the current study.

## Footnotes

1 In practice, in the health systems I am aware of, the decision here would be to provide the test to Rachel. This means that a health professional might feel that the ethical dilemma has been dissolved by the existence of a law, professional guideline, or shared expected practice. This doesn’t detract from the fact that there is something ethically difficult here, particularly within the context of a strongly patient-centred health system such as the UK.

2
https://www.bbc.co.uk/news/health-40554462 (Accessed 4th July 2025).
